# Link between History of Childhood Maltreatment and Emotion Dysregulation in Adults Suffering from Attention Deficit/Hyperactivity Disorder or Borderline Personality Disorder

**DOI:** 10.3390/biomedicines9101469

**Published:** 2021-10-14

**Authors:** Eva Rüfenacht, Eléonore Pham, Rosetta Nicastro, Karen Dieben, Roland Hasler, Sébastien Weibel, Nader Perroud

**Affiliations:** 1 TRE Unit, Division of Psychiatric Specialties, Department of Mental Health and Psychiatry, University Hospitals of Geneva, 1201 Geneva 4, Switzerland; eleonore.pham@hcuge.ch (E.P.); rosetta.nicastro@hcuge.ch (R.N.); karen.dieben@hcuge.ch (K.D.); Roland.Hasler@hcuge.ch (R.H.); Nader.Perroud@hcuge.ch (N.P.); 2 Department of Psychiatry, University Hospital of Strasbourg, 67091 Strasbourg, France; sebastien.weibel@chru-strasbourg.fr; 3 Physiopathologie et Psychopathologie Cognitive de la Schizophrénie—INSERM 1114, FMTS, University of Strasbourg, 67085 Strasbourg, France; 4 Department of Psychiatry, Faculty of Medicine, University of Geneva, 1211 Geneva 4, Switzerland

**Keywords:** childhood maltreatment, emotion dysregulation, attention deficit hyperactivity disorder, borderline personality disorder, comorbid attention deficit hyperactivity disorder and borderline personality disorder, cognitive emotion regulation strategies

## Abstract

Childhood maltreatment (CM) may have a long-term effect on emotion regulation. This study aimed to explore the relationship between CM and emotion dysregulation (ED) in a heterogeneous population. Four hundred seventy French-speaking outpatients (*N* = 279 ADHD, *N* = 70 BPD, *N* = 60 ADHD + BPD, *N* = 61 clinical controls) completed the Emotion Reactivity Scale (ERS), the Cognitive Emotional Regulation Questionnaire (CERQ), the Childhood Trauma Questionnaire (CTQ), and the Relationship Scales Questionnaire (RSQ). Reports of childhood maltreatment experiences were significantly associated with increased levels of emotion reactivity in all our groups and in the whole population, with a greater use of non-adaptive cognitive emotion regulation strategies and insecure attachment patterns. Emotional abuse showed the strongest effect. Further analysis indicated that an anxious attachment style significantly mediated the relationship between CM and the use of non-adaptive cognitive emotion regulation strategies and emotion reactivity. The results of our study suggest an impact of CM on ED and a potentially marked effect of emotional abuse. They also indicate a potentially mediating role of insecure attachment in the relationship between a history of childhood abuse and emotion reactivity and a higher use of non-adaptive cognitive emotion regulation strategies in adulthood.

## 1. Introduction

Experiencing childhood maltreatment can have a long-term impact on mental health and there is some evidence suggesting its association with several psychiatric disorders in adulthood [1,2,3,4,5,6,7,8,9,10,11,12,13]. In particular, previous research has found a strong relationship with childhood maltreatment and borderline personality disorder (BPD) [14,15,16,17]. Numerous BPD patients report past experiences of some form of childhood abuse, and individuals reporting a history of childhood abuse or neglect present a higher likelihood of developing a personality disorder (PD) during early adulthood [12,16,18,19,20]. Adverse childhood experiences have also been found to be correlated with BPD symptomatology severity [19,21]. Attention deficit/hyperactivity disorder (ADHD) is another psychiatric disorder for which data exist that support a link with childhood maltreatment [8,22,23]. ADHD appears to be frequent among maltreated children [23,24], and significantly higher rates of abuse are found in children with ADHD [25,26,27]. In adult populations, emotional abuse and emotional neglect have been found to be more frequent among patients with ADHD than in controls [9]. There is also some evidence suggesting that high levels of ADHD symptoms in adulthood may be associated with childhood maltreatment [8]. However, in adult ADHD populations, the results regarding a causal link between childhood abuse and adult ADHD are contradictory [8,26]. In summary, both BPD and, more recently, ADHD have been associated with childhood maltreatment [22,28,29,30,31].

One hypothesis concerning how adverse experiences may affect these two disorders is through their impact on emotion regulation. Emotion dysregulation (ED) is a transdiagnostic process known to be involved in several psychological disorders, particularly BPD, of which it is a core component [32,33]. Recent studies have also shown that ED may be considered as a cardinal symptom of ADHD in different age groups, including children, adolescents, and adults [34,35,36]. Childhood maltreatment can compromise the development of emotion regulation by its negative impact on different processes, including the use of adaptive emotion regulation strategies, emotion recognition, expression, and understanding [2,7,37,38,39,40]. Children who have suffered abuse seem to implement maladaptive emotion regulation strategies, such as emotion suppression, avoidance, and rumination, and continue to do so in adolescence [41,42,43]. Similarly, adults with a history of childhood maltreatment tend to use more maladaptive strategies such as experimental avoidance, expressive suppression, or emotional non-acceptance and fewer adaptive strategies such as reappraisal [2,44,45]. Furthermore, childhood abuse appears to be associated with higher emotional reactivity and expression of negative emotions in response to stress [43,46]. Specifically, maltreated children show more emotional lability, negativity, and inappropriate emotional displays and lower levels of empathy and self-awareness [38,41].

Childhood maltreatment may also hinder the development of mentalizing capacities [47,48,49,50]. The ability to mentalize depends on early attachment relationships and is thought to be an important developmental process that contributes to emotion regulation [48]. The impact of childhood abuse on attachment, and thus on the development of a capacity to mentalize, could play a central role in the emergence of emotion regulation difficulties later in life [51]. Childhood abuse is suggested to lead to insecure attachment patterns, such as anxious or avoidant attachments, which may further contribute to psychopathology development in a wide range of disorders [52,53,54,55,56]. This is particularly well established for BPD: previous work supports the presence of insecure attachment in BPD patients and suggests that core symptoms of the disorder may arise in relation to this aspect [57,58,59,60,61,62,63,64]. Similarly, although research is still scarce, there are some preliminary data suggesting a link between ADHD and insecure attachment [65,66]. In addition, several studies have explored how attachment patterns may influence the use of specific emotion regulation strategies [53,67]. In the literature, anxious attachment has been found to be associated with more intense negative emotional reactions and a tendency to ruminate and to struggle not to think about negative experiences; it contrasts with avoidant attachment, which is marked by suppression or avoidance of thoughts about negative experiences [67,68,69,70,71]. Additional data suggested that there is an association between anxious romantic attachment in adulthood and psychopathology, mediated by emotion-focused strategies, such as self-blame, rumination, and a focus on negative emotions [53]. Together, these observations contribute to our current understanding of possible mechanisms by which insecure attachment, related to childhood maltreatment, may impact mental health later in life. This can happen through difficulties with emotion regulation due to the use of specific emotional regulation strategies, and often results in poorer social functioning.

Results from our previous study [36] suggested that ADHD patients were more likely to use non-adaptive cognitive emotion regulation strategies than were healthy controls, and that, like BPD patients, non-adaptive cognitive emotion regulation strategies such as rumination played a role in emotion dysregulation in this patient group. In the present study, we specifically aimed to investigate the impact of early adverse experiences on emotion regulation in the same patient groups, namely BPD, ADHD, and BPD + ADHD patients. The first two populations were selected because of the key role emotion dysregulation plays in the clinical presentation, as well as the association of these disorders with childhood maltreatment. We added a BPD + ADHD group given the high level of co-occurrence of these disorders [30,72]. We also included a control population, consisting of patients referred for a diagnostic assessment of BPD and/or ADHD, but where the diagnosis was not confirmed. This clinical control group was added for comparison and to explore the specificity of the results in relation to the other clinical groups. Specifically, we were interested in measuring the impact of childhood maltreatment on emotion regulation strategies and emotion reactivity. We also wanted to assess the relation between attachment patterns and ED in our different groups. We hypothesized first that childhood traumatic experiences would be associated with ED, independent of the diagnostic category. Our second hypothesis was that insecure attachment should play a mediating role in the relationship between childhood maltreatment and ED.

## 2. Materials and Methods

### 2.1. Participants and Procedure

Four hundred and seventy French-speaking outpatients were recruited in a specialized center for diagnosis and treatment of adults suffering from ADHD and BPD at the University Hospitals of Geneva. Patients were initially referred to the center for a diagnostic assessment of these disorders.

The patients underwent a clinical evaluation conducted by a psychiatrist or psychologist trained in the assessment of ADHD and personality disorders to ascertain the diagnosis of BPD and/or ADHD and to exclude any organic condition and/or other psychiatric disorder that could better explain the symptoms. The evaluation was based on the Diagnostic Interview for ADHD in adults (DIVA 2.0), a structured diagnostic interview assessing DSM-IV ADHD criteria [73] (but for the purpose of this study, the DSM-5 criteria were applied, including the onset of symptoms before the age of 12 years and the presence of five criteria to meet the diagnosis in adulthood), and the Structured Clinical Interview for DSM-IV Axis II disorders (SCID-II) for BPD diagnosis [74].

ADHD symptomatology was assessed with the 25-item Wender Utah Rating Scale (WURS-25) [75] and the Adult ADHD Self-Report Scale, Version 1.1 (ASRS v1.1) [76], which are self-report questionnaires for the screening of childhood and adulthood ADHD, respectively. BPD symptomatology was assessed with the Borderline Symptom List (BSL-23) [77].

Two-hundred seventy-nine subjects were categorized as having ADHD, 70 as having BPD, and 60 as having both disorders (BPD + ADHD). Sixty-one patients referred to our specialized center were found, after a careful clinical assessment, not to have either of these disorders and were thus used as a clinical control group in our study. Note that 17 people (27.86%) in the control group reported experiencing symptoms suggestive of an ADHD diagnosis during their childhood but did not present the disorder as adults, in line with the observation that in the general population only roughly 15% of ADHD diagnoses persist in adulthood [78,79]. In addition, 16 people (26.23%) in the control group presented a history of major depressive disorder, 9 (14.75%) of anxiety disorder, 4 (6.56%) of eating disorder, and 3 (4.92%) of substance use disorder (Table 1).

The study was approved by the ethics committee of the University Hospitals of Geneva, and all subjects provided informed consent.

### 2.2. Assessment Instruments

The *Emotion Reactivity Scale* (ERS) [80] is a questionnaire inquiring about emotional experience. It is a 21-item self-report measure of emotion reactivity based on three aspects: emotion sensitivity, intensity, and persistence. Each item is rated on a scale from 1 to 4, with scores ranging from 0 to 40 for emotion sensitivity, 0 to 28 for emotion intensity, and 0 to 16 for persistence; thus, possible total scores range from 0 to 84.

The *Cognitive Emotional Regulation Questionnaire* (CERQ) [81] is a 36-item questionnaire consisting of nine conceptually different subscales based on different cognitive emotion regulation strategies, divided in two main groups: *adaptive strategies,* which comprise putting into perspective, positive refocusing, positive reappraisal, acceptance, and refocus on planning; and *non-adaptive strategies,* which comprise self-blame, blaming others, rumination, and catastrophizing. Each subscale contains four items referring to thoughts after the experience of threatening or stressful life events. The cognitive emotional regulation strategies are measured on a 5-point Likert scale, ranging from 1 (almost never) to 5 (almost always).

The *Childhood Trauma Questionnaire* (CTQ) [82] was used to assess history of childhood adversity. It consists of 28 items in five subscales measuring emotional, physical, and sexual abuse, as well as emotional and physical neglect.

The *Relationship Scales Questionnaire* (RSQ) [83] was used to measure attachment styles and participants’ feelings about close relationships. The RSQ is a 30-item questionnaire with one subscale for secure attachment and two subscales for insecure attachment: anxious and avoidant [84].

### 2.3. Statistics

All analyses were performed using Stata v16 (StataCorp LLC., College Station, TX, USA). Univariate comparisons between groups were conducted using the chi-square test (or Fisher’s exact test when the assumption of frequencies for the chi-square test was not met) for qualitative variables and a one-way ANOVA for quantitative variables.

Linear regression models with adjustments for age and gender were used to assess the effect of childhood maltreatment on scales assessing emotion reactivity, cognitive emotion regulation strategies, and attachment style in each clinical group separately. For analyses of the whole population, an additional adjustment for diagnostic group as the fixed effect was made. Statistical significance was accepted for *p*-values < 0.05.

A mediation analysis was used to assess the mediating effect of attachment style on the relationship between CTQ total score and emotion-related scales. Mediation analyses were only done in the whole population with adjustments for age, gender, and diagnostic group considering variables that showed a significant association with CTQ total score at a level of *p* < 0.001. Only total scores were considered. The methods described by Hicks and Tingley [85] using the “medeff” with 1000 simulations and 1000 bootstraps implemented in Stata v16 (StataCorp LLC., College Station, TX, USA) were applied.

## 3. Results

### 3.1. Demographic and Clinical Characteristics

The four groups (BPD, ADHD, BPD + ADHD, Controls) differed significantly in several demographic and clinical characteristics: age, gender, occupation, and lifetime comorbidities. Concerning scores on the different scales, the four groups differed significantly in almost all the scales except the CERQ acceptance subscale, the BES cognitive subscale, and the RSQ avoidance and secure subscales. The ADHD, BPD, and BPD + ADHD groups displayed significantly higher ED than did the Control subjects (b = 6.85, *p* = 0.009; b = 22.33, *p* < 0.001; and b = 26.12, *p* < 0.001, for the comparisons of ERS total scores between ADHD, BPD, and BPD + ADHD groups, respectively, vs. Controls). The BPD and BPD + ADHD samples (but not the ADHD group) used non-adaptive cognitive emotion strategies more often than did the Controls (b = 5.26, *p* = 0.006; and b = 7.9, *p* < 0.001, respectively) and adaptive cognitive strategies less often (b = −7.45, *p* = 0.005; and b = −6.27, *p* = 0.016, respectively). The BPD and BPD + ADHD groups, but not the ADHD group, displayed significantly higher levels of avoidant attachment (b = 3.41, *p* < 0.001; and b = 3, *p* < 0.001) than did the Controls.

### 3.2. Effect of Childhood Maltreatment on ERS Scores

Childhood maltreatment (total score) was significantly associated with higher ERS total scores in all our groups (ADHD: b = 0.18, *p* = 0.001; BPD: b = 0.21, *p* = 0.014; BPD + ADHD: b = 0.17, *p* = 0.013; Control: b = 0.57, *p* < 0.001). Considering the whole sample, childhood maltreatment also predicted higher ERS total scores (b = 0.23, *p* < 0.001) and higher scores on all three subscales (higher sensitivity, arousal, and persistence of emotion) (Table 2). In the whole sample, the strongest effect was found for emotional abuse on ERS total score (b = 0.22, *p* < 0.001) (Table 3).

### 3.3. Effect of Childhood Maltreatment on CERQ Scores

In the whole sample, looking at total scores on the CERQ, only non-adaptive strategies were significantly associated with childhood maltreatment (total score) (b = 0.25, *p* < 0.001; with b = −0.03, *p* = 0.472 for adaptive strategies) (Table 2). As with the ERS, the strongest effect on non-adaptive strategies was found for emotional abuse (b = 0.30, *p* < 0.001) (Table 3). All four non-adaptive strategy subscales were significantly associated with childhood maltreatment (total score) (Table 2).

Nevertheless, there were some differences between samples: the self-blame and rumination subscales were not significantly associated with childhood maltreatment in the ADHD and BPD + ADHD samples but were significantly associated in the BPD and Control groups, whereas the catastrophizing subscale was not significant in the BPD sample but was significant in the other ones.

Interestingly, among adaptive strategies, acceptance was significantly associated with childhood maltreatment (total score), with an effect mainly driven by the BPD sample in the sense that a higher level of childhood maltreatment was associated with higher acceptance (b = 0.37, *p* < 0.001) in this group. The other significant effect of childhood maltreatment (total score) (b = −0.10, *p* = 0.045) was on refocusing on planning, which is mainly driven by the ADHD sample in that a higher level of childhood maltreatment was associated with a lower refocusing on planning score (b = −0.22, *p* = 0.001).

### 3.4. Effect of Childhood Maltreatment on RSQ Scores

In the whole sample, childhood maltreatment (total score) was significantly associated with higher RSQ avoidant and anxious scores (b = 0.16, *p* = 0.002; and b = 0.27, *p* < 0.001, respectively) and lower RSQ secure scores (b = −0.11, *p* = 0.029) (Table 2). The strongest effect, which was found for the anxious attachment style, was observed in all samples, and was mainly explained by emotional abuse (b = 0.28, *p* < 0.001) (Table 3).

### 3.5. Mediation Analyses

**Figure 1:**
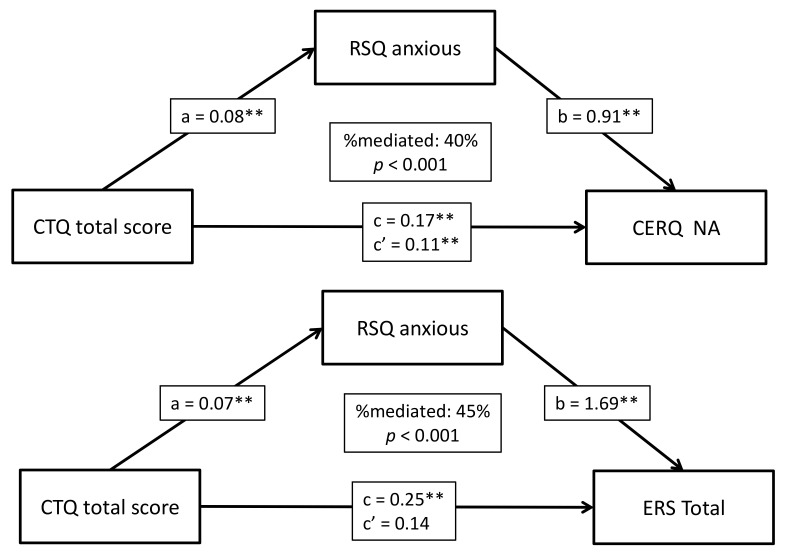
Mediation analyses in the whole population showing the effect of childhood maltreatment (CTQ total score) on CERQ non-adaptive strategies and ERS total score, mediated by anxious attachment (RSQ anxious). c: total effect of CTQ total score on outcomes (ab + c’); c’: direct effect of CTQ total score on outcomes; a: effect of the independent variable on the mediator; b: effect of the mediator on the dependent variable. ** *p* < 0.001.

For the strongest associations with the childhood maltreatment total score, namely the total scores for CERQ non-adaptive strategies and the ERS and the RSQ anxious scores, we wanted to investigate whether the associations between childhood maltreatment and the first two factors would be better explained by a mediating effect of anxious attachment. Indeed, we found a significant mediating effect of the RSQ anxious style on the association between the childhood maltreatment total score and the CERQ non-adaptive strategies and ERS total scores; at least 40% of the effect was mediated (*p* < 0.001) (Figure 1).

## 4. Discussion

Our study investigated whether childhood abuse could be linked to ED in a heterogenous sample including BPD, ADHD, and BPD + ADHD patients as well as a clinical control population consisting of patients referred for assessment for these disorders. We also wished to explore the use of certain cognitive emotional regulation strategies in relation to past experiences of childhood maltreatment. Finally, we investigated the potentially mediating role of insecure attachment on the relationship between childhood maltreatment and emotional dysregulation in adulthood.

The results of this study showed that childhood maltreatment, independent of clinical group, was associated with higher total scores on the scale measuring emotion reactivity. In the whole sample, this relationship also predicted higher values for all the dimensions constituting emotion reactivity, namely sensitivity, intensity, and persistence of the emotion. This observation extended to some degree to all groups individually, including our control group. These results suggest that, regardless of the diagnosis or the existence of a current psychiatric disorder, adverse childhood events may have a long-lasting impact on how individuals experience their emotions. A recent study showed similar results to ours: childhood maltreatment, especially emotional maltreatment, was positively associated with emotional regulation difficulties across different groups, namely healthy controls, individuals with BPD, and clinical controls consisting of individuals with ADHD and/or substance use disorder, without BPD [28]. Our study provided further evidence of an association between a history of childhood abuse and emotional symptoms in adulthood across a range of clinical and non-clinical populations [1,2,7,86,87,88].

Regarding the impact of different types of maltreatment, emotional abuse had the strongest effect on emotional reactivity in the total sample. There is some evidence in the literature supporting a particular association between emotional abuse in childhood and emotion regulation difficulties in adulthood, observed in several studies in different clinical and non-clinical populations, some of which explored features of BPD [19,28,89,90,91].

As for the different cognitive strategies used to regulate emotions, we found that all four non-adaptive strategy subscales (self-blame, rumination, blaming others, catastrophizing) were significantly associated with childhood maltreatment in the whole population. This result potentially suggests that individuals who have been exposed to adverse childhood experiences, regardless of their diagnosis or the existence of a current diagnosis, may use more non-adaptive cognitive emotion regulation strategies than adaptive ones. There is current evidence in the literature suggesting that exposure to childhood trauma may have a deleterious effect on the strategies used to regulate emotions in different age groups, which may be maladaptive, including greater use of rumination and expressive suppression [43,45,46,92,93,94].

Interestingly, in our study, emotional abuse presented the strongest association with the use of non-adaptive cognitive emotion regulation strategies. Similar results were found in a study comparing BPD, major depressive disorder, and healthy control populations, where a history of emotional abuse was associated with dysfunctional emotion regulation strategies, in this case expressive suppression [44]. Past experiences of emotional neglect were linked to decreased use of adaptive strategies such as cognitive reappraisal [44]. Additional data from a non-clinical sample suggested that a history of childhood emotional invalidation was associated with chronic emotional inhibition in adulthood (e.g., ambivalence over emotional expression, thought suppression, and avoidant stress responses), which in turn significantly predicted psychological distress [95]. Overall, there is a considerable amount of data in the literature that suggest a detrimental impact of childhood maltreatment, and specifically emotional abuse, on the development of emotion regulation strategies.

The last part of our data analysis focused on exploring the association between total score for childhood maltreatment and attachment patterns in the whole sample. Our results showed that childhood maltreatment predicted higher scores for insecure attachment styles and lower scores for secure attachment. In the literature on the topic, childhood maltreatment is associated with insecure patterns of attachment that may persist in adulthood [96,97,98,99,100]. There is evidence in favor of a long-term impact of insecure attachment, which contributes to emotional regulation difficulties and the development of psychopathology in adulthood [53,54,97,101,102,103,104]. Both insecure attachment styles were found to increase vulnerability when dealing with life’s adversities and the risk of distress maintenance and psychopathology development over time [53,54,97,105].

When we further explored the strongest and most significant associations with childhood maltreatment total score, which included the CERQ non-adaptive strategies total score, the ERS total score, and the RSQ anxious style score, we found a significant mediating effect of the RSQ anxious style on the association between childhood maltreatment total score and CERQ non-adaptive strategies and ERS total scores: at least 40% of the effect was mediated (*p* < 0.001). There are some data in the literature suggesting a mediating role of insecure attachment in the development of psychopathology after a history of childhood abuse. A previous prospective study observed that insecure attachment style played a mediating role in the relationship between childhood maltreatment and depressive symptoms in young adulthood [106]. Further data highlighted the role of the anxious attachment style in adulthood in partially explaining the relationship between childhood neglect and physical abuse, and later depression and anxiety symptoms [100]. One potential explanation for this observation may be that insecure attachment patterns can lead to the development of psychopathology, indirectly and potentially through emotion regulation strategies [53,54]. Insecure attachment styles may predispose individuals to function with certain patterns when relating to themselves and others, which in turn may influence the use of specific emotion regulation strategies when facing challenges [54,104]. The use of these strategies may not be helpful in resolving difficulties and could promote further distress, while contributing to emotion dysregulation [54,104]. The avoidant style is described as separating emotions from thoughts and actions, while suppressing the experience of distress [53,54,68]. Individuals who present with an anxious attachment style experience more negative emotions through hyperactivation of their attachment system, favoring emotion amplification and exaggeration of worries [53,54,67]. Moreover, they may make greater use of emotion regulation strategies such as rumination and self-blame [53,54,67]. Our results partly support the literature on the topic, as anxious attachment was found to mediate greater use of non-adaptive cognitive emotion regulation strategies and increased emotion reactivity in a population with a history of childhood abuse.

Our study has several limitations. The first relates to the use of a cross-sectional design, with retrospective assessments of childhood abuse through self-report questionnaires, which may induce some bias. Further to this, using a mediation analysis in this setting also limits the possibility to infer any causal effects, and leaves uncertainty on the direction of the mediation. Our analysis can only draw hypotheses that may potentially be explored further in prospective studies. One could also question whether our clinical control group might have been affected by selection bias, as the individuals may have been referred in some cases to the clinical service because of emotion regulation difficulties or have presented sub-threshold BPD or ADHD features. There is evidence supporting the clinical relevance of subthreshold symptoms [107,108]. Besides this, some of the clinical features for these diagnoses might have also gone unrecognized during childhood and adolescence, which could in turn influence the data collected regarding earlier comorbidities [109]. Further to this observation, our study did not include a non-clinical group that would have allowed us to examine the non-specificity of results to a greater extent. Additionally, there was also a notable difference in sample size, with the ADHD group presenting the highest number of participants. This can be explained by our decision to include all potential subjects in our study, although this may add a bias and impact on our results. Another point worth mentioning concerns our analyses. We performed a large number of tests and in an effort to be exhaustive all results with a *p*-value below 0.05 were reported as significant, which could possibly overestimate the significance of our results. Given the exploratory nature of our analysis, the *p*-value was set to <0.05. In order to account for multiple testing, the following can be applied for the association between CTQ and self-report scales: (3 (scales: ERS, CERQ and RSQ) * 4 (populations: ADHD, BPD, BPD + ADHD and Control) = 0.05/12 = 0.004). Due to the high collinearity between subscales in each scale, only one test was considered for each of the scales. The *p*-value of 0.004 could then be used as a threshold to only consider the most relevant of our results. We also did not explore individual non-adaptive strategies in relation to abuse types in more detail. One final limitation on the comparison of our results with some of the previously published data may be the choice of measures we used for attachment patterns and emotional difficulties, as they may vary throughout studies. While taking these different observations into account, our results may provide some additional data on the impact of childhood abuse, and specifically emotional abuse, on emotion regulation difficulties later in life.

## 5. Conclusions

The results of our study back up previous data suggesting that childhood maltreatment has an impact on emotion dysregulation, and that the effect of emotional abuse can potentially be marked. Reports of past childhood abuse were associated with increased levels of emotion reactivity in all our groups and higher use of non-adaptive strategies in the whole sample, with emotional abuse having the strongest effect. Our data also suggest that insecure attachment has a mediating role in the relationship between a history of abuse and emotion dysregulation in adulthood. In the future, we intend to explore these observations further and add other dimensions that play a role in emotion regulation, such as reflective functioning. We are also interested in investigating the non-specific impact of childhood abuse on emotion dysregulation in more detail, and determine whether this transdiagnostic process may result from other common underlying difficulties in different patient groups.

Our findings also highlight the importance of several considerations that could be useful in clinical practice. The first one is the relevance of enquiring about a history of childhood abuse when conducting clinical assessment of patients presenting with emotion regulation difficulties. The significant impact of emotional abuse emphasizes the importance of exploring a history of emotional abuse when conducting assessments, and not solely taking into consideration experiences of sexual or physical maltreatment. Furthermore, interventions designed to increase the use of adaptive cognitive emotion regulation strategies and reduce non-adaptive ones would be particularly helpful treatment targets to improve emotion regulation in patients with a history of childhood abuse.

## Figures and Tables

**Table 1:** Clinical and demographic characteristics of participants.

		ADHD		BPD		BPD + ADHD		Cont.		
		*N* = 279		*N* = 70		*N* = 60		*N* = 61		
		Mean or N	SD or %	Mean or N	SD or %	Mean or N	SD or %	Mean or N	SD or %	*p*
Age		35.49	12.86	31.66	9.08	30.29	8.64	35.24	12.29	0.005
Gender	Female	122	43.73	66	94.29	55	91.67	34	55.74	<0.001
Civil status *	Single (vs. not single)	130	49.24	36	51.43	33	57.89	31	52.54	0.84
Children *	0	163	61.74	46	65.71	43	74.14	39	66.1	0.11
	1	28	10.61	13	18.57	7	12.07	6	10.17	
	≥2	73	27.65	11	15.71	8	13.79	14	23.73	
Job *	Yes (vs. no)	157	61.33	30	42.86	26	48.15	27	45.76	0.026
Years of education *		15.52	2.86	15.23	3.18	14.87	2.69	15.76	3.2	0.236
Lifetime comorbidities	Major depressive disorder	126	45.16	58	82.86	42	70	16	26.23	<0.001
	Bipolar disorder	6	2.15	7	10	6	10	0	0	0.001
	Anxiety disorder **	57	20.43	58	82.86	33	55	9	14.75	<0.001
	Eating disorders	10	3.58	15	21.43	11	18.33	4	6.56	<0.001
	Substance use disorder	71	25.45	24	34.29	24	40	3	4.92	<0.001
ERS	Sensitivity	22.31	8.78	29.23	7.77	31.26	5.95	19.49	9.36	<0.001
	Arousal/Intensity	15.84	7.06	22.07	5.65	22.86	4.49	13.07	7.39	<0.001
	Persistence	9.23	3.78	11.71	3.26	12.5	2.7	8.13	3.91	<0.001
	Total	47.38	18.39	63.01	15.61	66.61	12.1	40.69	19.63	<0.001
CERQ	Self-blame	11.53	3.75	13.82	3.56	14.06	3.6	11.88	4.23	<0.001
	Acceptance	12.83	3.38	13.12	3.46	13.38	3.44	13.38	3.60	0.578
	Rumination	12.94	3.86	14.36	3.87	14.79	3.54	13.09	3.92	0.001
	Positive refocusing	9	3.68	7.71	3.63	7.39	3.17	8.75	4.10	0.004
	Refocusing on planning	12.94	3.63	10.82	3.93	11.65	3.53	13.42	3.72	<0.001
	Positive reappraisal	12.45	3.89	10.07	4.12	10.53	3.56	12.17	4.27	<0.001
	Putting into perspective	12.27	3.82	10.76	3.83	10.81	3.66	12.32	3.93	0.002
	Catastrophizing	8.66	3.6	10.27	3.86	10.79	3.93	8.88	3.47	<0.001
	Blaming others	8.84	3.53	9.49	3.88	10.61	3.6	8.83	3.54	0.001
	Total: Adaptive strategies	59.49	13.29	52.58	14.02	53.76	11.86	60.03	15.60	<0.001
	Total: Non-adaptive strategies	41.97	10.66	47.93	10.8	50.57	10.32	42.67	10.78	<0.001
RSQ	Avoidant	21.89	4.19	22.49	4.34	22.30	4.18	22.47	3.62	0.59
	Anxious	13.80	4.13	17.37	4.23	16.96	3.13	13.95	4.18	<0.001
	Secure	16.81	3.06	16.55	3.70	17.64	3.06	16.87	2.94	0.25
CTQ	Emotional abuse	10.89	5.33	15.18	5.60	16.03	5.88	10.25	5.16	<0.001
	Physical abuse	7.03	3.38	8.57	4.92	9.01	5.25	6.20	2.04	<0.001
	Sexual abuse	5.91	2.76	9.02	6.23	8.55	6.05	6.62	3.69	<0.001
	Emotional neglect	12.79	4.94	15.30	4.63	15.43	5.33	12.03	5.09	<0.001
	Physical neglect	7.82	3.03	9.20	3.92	9.08	4.30	7.84	3.10	0.003
	Total	44.20	14.73	56.16	20.75	58.09	20.13	42.95	14.49	<0.001

* Missing values encountered; ** Including generalized anxiety disorder, panic disorder, social phobia, and obsessive-compulsive disorder.

**Table 2:** Association between CTQ total score and ERS, CERQ, and RSQ questionnaires for ADHD, BPD, BPD + ADHD, and Control groups and the whole population (All).

		ADHD		BPD		BPD + ADHD		Control		All	
		b	*p*	b	*p*	b	*p*	b	*p*	b	*p*
ERS	Sensitivity	0.17	0.012	0.22	0.018	0.17	0.022	0.54	<0.001	0.21	<0.001
	Arousal/intensity	0.19	0.004	0.21	0.013	0.15	0.032	0.53	<0.001	0.21	<0.001
	Persistence	0.11	0.108	0.16	0.075	0.19	0.014	0.57	<0.001	0.17	<0.001
	Total	0.18	0.001	0.21	0.014	0.17	0.013	0.57	<0.001	0.23	<0.001
CERQ	Self-blame	0.05	0.445	0.24	0.012	0.07	0.494	0.43	0.023	0.13	0.007
	Acceptance	0.1	0.158	0.37	<0.001	0.19	0.07	0.05	0.798	0.18	<0.001
	Rumination	0.03	0.379	0.25	0.021	0.08	0.428	0.5	0.002	0.14	0.005
	Positive refocusing	−0.04	0.574	−0.07	0.601	−0.09	0.314	−0.4	0.039	−0.09	0.087
	Refocusing on planning	−0.22	0.001	0.08	0.394	0.03	0.574	−0.29	0.086	−0.1	0.045
	Positive reappraisal	−0.09	0.182	0.01	0.931	0.03	0.762	−0.38	0.043	−0.07	0.167
	Putting into perspective	−0.11	0.11	0.12	0.235	−0.01	0.997	−0.17	0.329	−0.04	0.451
	Catastrophizing	0.17	0.018	0.11	0.306	0.41	<0.001	0.4	0.011	0.21	<0.001
	Blaming others	0.14	0.04	0.29	0.009	0.33	0.024	0.33	0.04	0.23	<0.001
	Total: Adaptive strategies	−0.11	0.129	0.13	0.236	0.04	0.664	−0.34	0.091	−0.03	0.472
	Total: Non-adaptive strategies	0.14	0.05	0.31	0.003	0.31	0.002	0.58	<0.001	0.25	<0.001
RSQ	Avoidant	0.17	0.025	0.16	0.161	0.01	0.932	0.37	0.014	0.16	0.002
	Anxious	0.27	<0.001	0.24	0.016	0.22	0.022	0.36	0.03	0.27	<0.001
	Secure	−0.09	0.192	−0.16	0.19	−0.15	0.25	−0.12	0.464	−0.11	0.029

**Table 3:** Effect of different types of childhood maltreatment on ERS total score, CERQ adaptive and non-adaptive strategies total scores, and RSQ avoidant, anxious, and secure subscales.

		Emotional Abuse		Emotional Neglect		Physical Abuse		Physical Neglect		Sexual Abuse	
		*b*	*p*	*b*	*p*	*b*	*p*	*b*	*p*	*b*	*p*
ERS	Total	0.22	<0.001	0.15	0.001	0.14	0.001	0.11	0.009	0.12	0.004
CERQ	Total–Adaptive strategies	−0.02	0.673	−0.17	<0.001	0.03	0.519	0.02	0.597	0.08	0.081
	Total–Non-adaptive strategies	0.3	<0.001	0.16	0.001	0.18	<0.001	0.13	0.005	0.16	0.001
RSQ	Avoidant	0.18	<0.001	0.17	0.001	0.06	0.191	0.16	0.001	0.1	0.038
	Anxious	0.28	<0.001	0.23	<0.001	0.16	0.001	0.17	<0.001	0.11	0.02
	Secure	−0.01	0.706	−0.13	0.009	−0.03	0.533	−0.12	0.012	−0.05	0.271

## Data Availability

The datasets used and/or analyzed during the current study are available from the corresponding author on reasonable request.
